# Pregnancy complicated with aortic dissection: Two cases report

**DOI:** 10.1097/MD.0000000000031487

**Published:** 2022-11-04

**Authors:** Xiaoli Wang, Xi Liu, E. Liao, Ning Ge, Yaping Hu

**Affiliations:** a Department of Obstetrics, Maternal and Child Health Hospital of Hubei Province, Tongji Medical College, Huazhong University of Science and Technology, Wuhan, China; b Radiology Department, Maternal and Child Health Hospital of Hubei Province, Tongji Medical College, Huazhong University of Science and Technology, Wuhan, China.

**Keywords:** aortic dissection, back pain, hypertension, pregnancy

## Abstract

This study aims to investigate the characteristics of pregnancy complicated with aortic dissection (AD), diagnosis and treatment plan, and maternal and infant outcomes.

**Patient concerns:**

Two pregnant women suffered persistent back pain were admitted to Hubei Maternal and Child Health Hospital from December 2019 to December 2020.

**Diagnosis:**

Pregnant women with chest and back pain and especially hypertension should be highly suspected of AD. However, to confirm diagnosis results, laboratory tests such as D-dimer, fibrinogen and white blood cells, and even some Special examination, cardiac ultrasound, computed tomographic angiography (CTA), magnetic resonance angiography (MRA), are required. Early diagnosis and intervention can improve maternal and infant outcomes.

**Interventions:**

Cesarean sections were performed in both patients. Case 1 underwent thoracic aortic stent implantation one day after the onset of AD symptoms. Case 2 received endovascular repair of AD 4 days after the onset of AD symptoms.

**Outcomes:**

In these two cases, good maternal and infant outcomes were obtained through effective early identification and treatment.

**Lessons:**

AD is characterized with an acute onset, and the rates of misdiagnosis and missed diagnosis are high, which seriously endangers the life of mother and child. Hypertension is one of the high-risk factors causing AD. Good maternal and infant outcomes can be achieved by early identification, multidisciplinary collaboration and timely cardiac surgical intervention.

## 1. Introduction

Aortic dissection (AD) during pregnancy is a rare macrovascular disease that threatens the mother and the fetus. It can occur at any stage of pregnancy, especially more common in the third trimester and puerperium. The onset of AD is acute and its treatment is difficult. Blood perfuses into the arterial wall from the tear opening to form a false lumen, which divides the aortic lumen into true lumen and false lumen, causing arterial rupture and hemorrhage. The morbidity of AD is 55/10,000.^[1]^ This study retrospectively analyzed the clinical data of 2 cases of pregnancy complicated with AD by reviewing the relevant literature in order to provide a basis for obstetric clinicians to understand, early identify and diagnose the disease.

## 2. Case presentation

**Figure 1.**
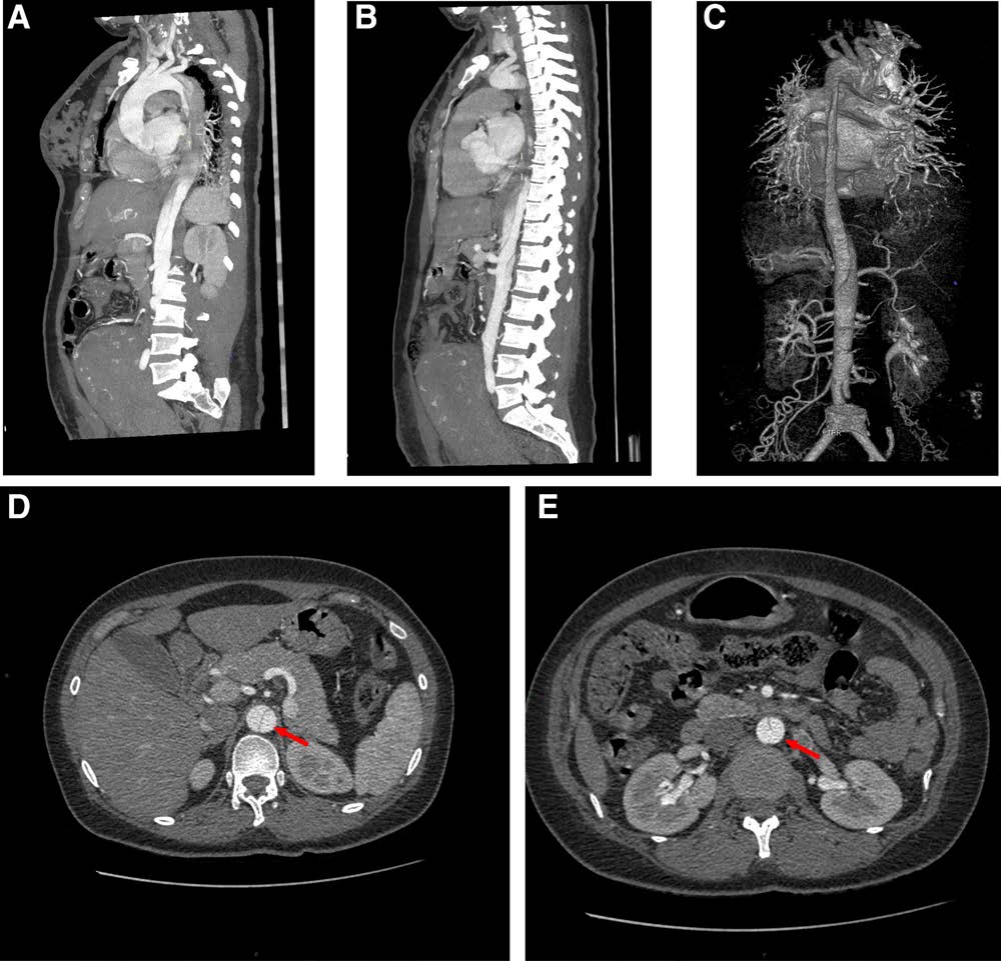
Type Debakey III, Type Stanford B: the celiac trunk, superior and inferior mesenteric arteries and left renal artery open from the true lumen, while the right renal artery seems to span the true and false lumen. (A) A long filling defect in the distal lumen of the initial part of the left subclavian artery. (B and C) True and false lumen development in the abdominal aorta from the lower diaphragm to the bifurcation. (D) Inward migration of inner diaphragm. (E) The location of the breach is at L1 and L3 level.

Case 1: A 38-year-old female patient was admitted to the hospital at gestational age of 38 + 3 weeks due to elevated blood sugar for 2 months and elevated blood pressure for 15 days. She had abnormal blood sugar during pregnancy and accepted diet. The blood pressure in the third trimester was up to 157/75 mm Hg. She had a history of myomectomy. The blood pressure on admission was 143/79 mm Hg, and body mass index was 23.2. A cesarean section was performed due to “uterine scar,” and the highest postoperative blood pressure was 141/97 mm Hg. On the third day after the operation, chest tightness occurred, accompanied by paroxysmal pain in the upper abdomen and back, tear-like pain in the shoulder and back, as well as nausea and retching. Physical examination showed blood pressure of 132/87 mm Hg, tenderness and rebound tenderness under the xiphoid process (tenderness at the lower edge of the spine flattened to the scapula), and positive right rib. Multidisciplinary consultation involving critical care medicine department, cardiology department, anesthesiology department was conducted immediately. Computed tomographic angiography (CTA) (Fig. 1) shows AD (Debakey type III). D-dimer is 15.9 μg/mL↑ (<0.5 μg/mL). She was transferred to a general hospital and underwent “thoracic aortic stent implantation” 4 days after the operation.

**Figure 2.**
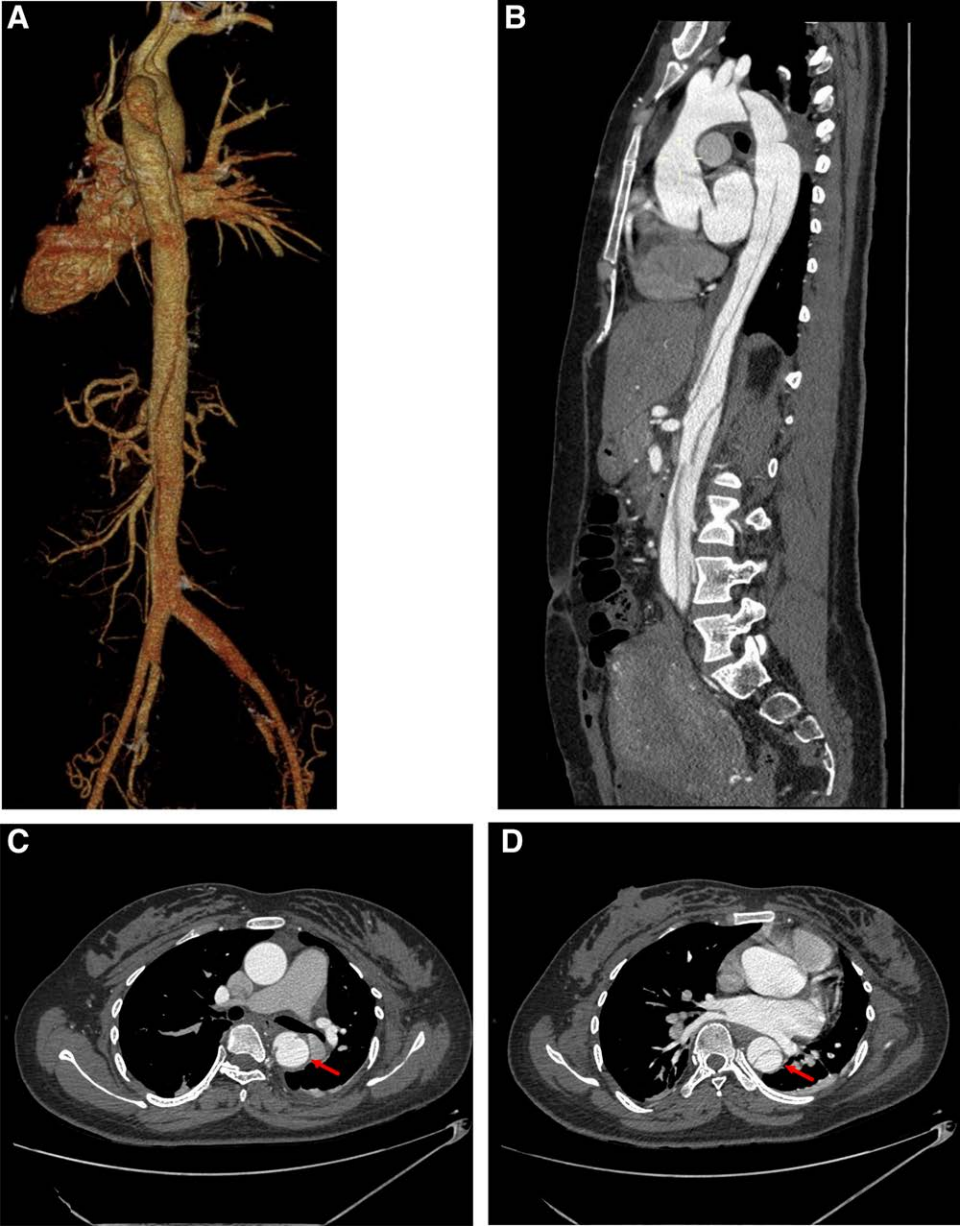
Type Debakey III, Type Stanford B: celiac trunk, superior mesenteric artery, inferior mesenteric artery and left renal artery open from the true lumen, and the right renal artery seems to cross the true and false lumen. (A and B) True and false lumen development can be seen in the lumen of aortic arch, descending aorta and bilateral iliac arteries. (C) the location of the break is at the level of the T6-7 vertebral body. (D) displacement of inner diaphragm.

Case 2: A 27-year-old female patient was admitted to the hospital at gestational age of 37 + 3 weeks due to abnormal fetal heart. The highest blood pressure in the third trimester was 133/90 mm Hg; the blood pressure on admission was 142/90 mm Hg, and the body mass index was 21.4. Check urine protein+. One hour after admission, persistent back pain occured, which was aggravated, accompanied by paroxysmal and profuse sweating. Blood pressure was 148/92 mm Hg. D-dimer was 6.98 μg/mL↑ (<0.5 μg/mL). Electrocardiogram (ECG), chest X-ray, echocardiography showed no abnormalities. In addition, multidisciplinary consultation involving intensive medicine, anesthesiology, cardiology was carried out, indicating that AD was highly suspected, and emergency cesarean section was performed to save the lives of pregnant women and fetuses. Intraoperative blood pressure was 130-200/60-100 mm Hg, and postoperative blood pressure was 180/90 mm Hg. Two hours after operation, the blood oxygen was 86%, which reached 95% to 99% after application of mask oxygen. CTA (Fig. 2) indicated AD (Debakey type III). She was transferred to a general hospital, and underwent “endovascular repair of aortic dissection” 4 days after the operation.

## 3. Materials and methods

Case selection: Two cases of pregnancy complicated with AD, aged 27 to 38 years old, admitted to Hubei Maternal and Child Health Hospital from December 2019 to December 2020 were selected, including one in the third trimester and the other one in the puerperium. The main symptoms were tear-like pain in the chest and back.

Methods: The clinical data of the selected 2 cases were retrospectively analyzed, including general data (Table 1: age, parity, past history, surgical history, clinical manifestations), auxiliary examinations (Table 2: ECG, transthoracic echocardiography, aortic blood vessels, angiography, MRI), classification of AD, laboratory parameters (Table 3: D-dimer, fibrinogen, leukocytes), treatment (surgical method, timing of surgery), and maternal and fetal outcomes.

**Table 1:** General information of 2 cases of pregnancy complicated with aortic dissection.

Number	Pregnancy	Age (yrs)	Bloodpressur (mm Hg)	Symptom	Previous history	AD classification	Diagnosis
1	G2P1 multipara	38	121–141/87–97	Chest and back pain	Myomectomy	DebakeyIII, Stanford B	TTE and MRA
2	G3P1 multipara; One previous abortion	27	130–200/60–100	Back pain	No special medical history	DebakeyIII, Stanford B	CTA

CTA = computed tomographic angiography, MRA = magnetic resonance angiography.

**Table 2:** Special examination of 2 cases of pregnancy complicated with aortic dissection.

Number	ECG	TTE	Chest X-ray	CTA or MRA	Treatment	Fate
1	No abnormal	Not done	Not done	Stanford B	Thoracic aortic stenting	Good
2	No abnormal	No abnormal	No abnormal	Stanford B	Endovascular repair of aortic dissection	Good

CTA = computed tomographic angiography, ECG = electrocardiogram, MRA = magnetic resonance angiography, TTE = transesophageal echocardiography.

**Table 3:** Laboratory examinations of 2 cases of pregnancy complicated with aortic dissection.

Number	D-dimer (<0.5 μg/mL)	FIB (2.38~4.98g/L)	Leukocyte (3.5~15 × 10^9^)
1	15.9 ↑	5.68 ↑	9.86
2	6.98 ↑	3.44	11.19

FIB = fibrinogen.

According to the origin of the dissection and the site of aortic involvement, there are two classifications. Stanford classification: regardless of the origin, all types involving the ascending aorta are type A (equivalent to Debakey types I and II), requiring active surgical treatment, and the prognosis is poor; type B dissection is limited to the descending aorta (Equivalent to DeBakey type III), which can be treated with surgery or drugs. Among the pregnant women complicated with AD, type A accounts for 50% to 89%, with the prehospital mortality rate as high as 53%, while type B accounts for 11% to 21%, with better prognosis than type A.^[1]^

## 4. Result

Of the 2 patients, one case was primipara, and the other 1 case was multiparous; 1 case was in the third trimester, and the other 1 was in the puerperium; 1 case was gestational hypertension, and the other 1 was severe preeclampsia; tear-like pain was the common main manifestation, as shown in Table 1. Related laboratory indicators indicate that D-dimer was significantly increased, as shown in Table 3. The AD by aortic angiography or magnetic resonance imaging was classified as Stanford B type, as shown in Figures 1 and 2.

### 4.1. Treatment and prognosis of mother and fetus

Both patients underwent aortic surgery and both survived with good prognosis. For the 1 in the third trimester, endovascular repair of AD was performed 4 days after emergency cesarean section; for the 1 in the puerperium, thoracic aortic stent implantation was performed 4 days after operation. Both neonates had good birth scores with no adverse outcomes.

## 5. Discussion

### 5.1. *Early identification of high-risk factors for* AD *in pregnancy*

Early identification of risk factors and underlying diseases associated with AD in pregnancy can improve the prognosis of the patients.^[2]^ The etiology of AD is still unclear, and in fact it is considered to be related to multiple factors. The risk factors for its pathogenesis include congenital factors and acquired factors. Further, congenital factors include certain hereditary connective tissue diseases, such as MFS (particularly common), Ehlers-Danlos syndrome, Loeys-Dietz syndrome, bone aneurysm syndrome, bilobal aortic valve malformation and aortic stenosis,^[3–6]^ acquired factors include hypertension, advanced age, iatrogenic injury, pregnancy, atherosclerosis, etc.^[3,4,6]^ In this study, both patients had gestational hypertension, which further supports the high-risk factors of pregnancy complicated with AD reported in the literature.

Pregnancy and childbirth are independent high-risk factors for AD. Due to rapid onset, rapid development, and extremely high mortality of AD, the identification of high-risk factors is of great importance. According to relevant literature reports, changes in cardiac hemodynamics and hormone levels during pregnancy lead to higher possibility of AD in pregnancy. AD can occur at any stage of pregnancy, but it is more common in the third trimester and puerperium.^[7]^ Studies have found that high levels of estrogen and progesterone can inhibit the deposition of collagen and elastic fibers in the aortic wall, promote the deposition of non-collagen proteins, and degenerate the blood vessel wall, thereby accelerating the formation of AD and increasing the rupture of the aorta. risk.^[8]^ Japanese scholars Tanaka et al^[9]^ pointed out that a large amount of blood refluxes and interstitial fluid were reabsorbed into the systemic circulation after childbirth. The total blood volume of the puerpera within 72 hours after childbirth was increased by 25% to 30% compared with the non-pregnant period, and the left ventricular stroke volume increased by about 33%, the impact force of blood flow on the aortic wall increased. During pregnancy, as the uterus gradually increases, the aorta is compressed and the pressure on the aortic wall increases. These changes increase the shear stress of blood flow on the blood vessel wall and aggravate the damage to the blood vessel wall, resulting in AD.^[10–12]^

### 5.2. Early diagnosis of pregnancy complicated with AD

AD is mainly diagnosed by clinical manifestations, signs, laboratory examinations, and imaging examinations. Pain is usually the most significant symptom of AD, which is manifested as persistent tear-like severe pain in the head, neck, chest, back, waist, abdomen or lower extremities, and is often difficult to relieve with strong analgesics such as morphine and pethidine. Most patients have hypertension at the time of onset, and asymmetry of arterial blood pressure in bilateral extremities is also an important manifestation.^[13]^ In this study, both patients had the above symptoms, and their symptoms were easily misdiagnosed as pulmonary embolism, coronary heart disease, and acute abdomen. When pregnant women have acute chest pain (70%), back pain (21.7%) or epigastric pain (2.5%), as well as dyspnea, persistent cough, syncope, and circulatory failure, the possibility of AD should be considered.^[3]^

Therefore, further laboratory tests and imaging studies are required. According to reports, white blood cell count and neutrophil count are often increased in the blood routine examination of pregnancy with AD, which may be related to the infiltration of lymphocytes and macrophages in the medial layer of the aortic wall and the adventitia of the blood vessel wall at the onset of AD. Therefore, when diagnosing pregnancy with AD, attention should be paid to the results of routine blood tests of pregnant women.

D-dimer is a degradation product of cross-linked fibrin. AD leads to damage to the aortic wall, then exposure of vascular coagulation activator and release of tissue factor activates the fibrinolytic system, causing an imbalance between the coagulation system and the fibrinolytic system, resulting in increased D-dimer levels. In addition to AD, acute coronary syndrome, acute pulmonary embolism, and acute myocardial infarction also show elevated plasma D-dimer levels. However, the ECG, myocardial enzymes and other laboratory test results of the above diseases have certain specific manifestations, which can be associated with phase identification of AD.^[14]^ For the 2 cases in this study, no obvious abnormality was found in the relevant laboratory indicators such as ECG and myocardial enzymes. D-dimer levels and fibrin degradation products in pregnant women were significantly increased when they were treated, indicating that pregnant women may have AD and hematoma.^[15]^ Therefore, D-dimer can be used as an early screening index for pregnancy complicated with AD.^[16]^ In this study, the level of D-dimer was significantly increased, which was consistent with the relevant literature reports.

For the selection of imaging examination methods for pregnant women complicated with AD, transthoracic echocardiography (TTE) can be used as a preliminary screening method considering its high safety and technical popularity. Gao Shuang et al^[17]^ retrospectively analyzed the data of 16 pregnant patients with AD and found that the detection rate of the disease by transthoracic echocardiography was 100%, the classification accuracy was 100%, and the endometrium located by ultrasound was 100%. The site of tear was consistent with angiography (CTA) findings and/or intraoperative findings. This test can provide clues for early screening and early diagnosis of pregnancy complicated with AD and has high clinical application value. However, one case in this study did not undergo echocardiography, and the other one showed no abnormality in echocardiography, which is inconsistent with the relevant literature reports, which may be related to the small number of cases in this study.

Patients with indeterminate diagnosis by TTE or with high suspicion of AD are recommended to undergo further whole-aortic CTA or magnetic resonance angiography (MRA). The sensitivity and specificity of thoracoabdominal aortic angiography (CTA) for AD is as high as 100%. Since CTA requires the use of a contrast agent, it is necessary to evaluate the renal function of the pregnant woman and judge whether she is allergic to the contrast agent before the examination.

MRA has the same effect as CTA, and its advantage is that there is no influence of ionizing radiation and contrast agents. Non-enhanced MRA is used to quickly assess the size of the aorta during pregnancy. Relevant experimental data^[18]^ have showed that this method is safer for pregnant women. However, MRA examinations take a long time and may be unbearable for patients with severe pain.

After finishing the diagnosis of AD by the above examinations, multidisciplinary consultation should be initiated immediately, and the patient should be immediately transferred to a center with cardiovascular treatment capabilities to improve the outcomes of mother and fetus.

### 5.3. Treatment of pregnancy complicated with AD and timing of pregnancy termination

The obstetric management of pregnancy complicated with AD should be comprehensively evaluated according to the actual situations of the pregnant woman and the fetus. In the past, many domestic advocate immediate surgical treatment, termination of pregnancy in the early stage of pregnancy, surgical repair of dissection before 28 weeks to continue pregnancy, close monitoring of fetal intrauterine conditions, and prolonging gestational weeks as much as possible. After 32 weeks of pregnancy, the fetus is basically matured, and the pregnancy can be terminated by cesarean section first followed by surgery.^[19,20]^ The prognosis of patients with onset in the second trimester is often poor. Since the disease itself will limit fetal growth or even cause intrauterine fetal death, patients with late onset tend to have a better prognosis.^[21]^ In this study, the 2 patients had onset in the third trimester, and the outcomes of the mother and fetus were good through timely treatment.

In conclusion, pregnancy complicated with AD is a very rare emergency in obstetrics, and early identification, early diagnosis, and early treatment are particularly important. Due to the lack of specificity in clinical symptoms and signs, we need to be strictly vigilant for the pregnant women with chest and back pain, elevated D-polymer level, fibrinogen degradation products, hypertension, vascular disease and connective tissue disease. Complete examination of CTA and MRA is recommended. Once AD is diagnosed, blood pressure and heart rate should be actively controlled, multidisciplinary consultation should be initiated immediately, and pregnant women should be transferred to medical institutions with vascular surgery centers. The medical institution of this study is a tertiary specialized hospital. Early identification and diagnosis of AD are particularly important, in order to save time for further referral and treatment and to ensure the safety of mothers and babies. There were 2 cases in this study, including 1 case in the third trimester, whose onset was sudden, treatment was quick, diagnosis was rapid, and good maternal and infant outcomes were obtained.

Under the multidisciplinary cooperation, a management model of active prevention, management during pregnancy, early identification, early diagnosis and effective treatment is gradually formed, which is an important strategy for pregnancy complicated with AD.

## Author contributions

**Conceptualization:** Yaping Hu.

**Data curation:** Xiaoli Wang, Xi Liu, Ning Ge.

**Formal analysis:** Xiaoli Wang, Xi Liu, Ning Ge.

**Investigation:** Xi Liu, Yaping Hu.

**Methodology:** Xi Liu, Yaping Hu.

**Project administration:** Yaping Hu.

**Resources:** Xi Liu, E. Liao.

**Software:** E. Liao, Ning Ge.

**Writing – original draft:** Xiaoli Wang.

**Writing – review & editing:** Xi Liu, Yaping Hu.
